# Multifaceted Interaction Between Hepatitis B Virus Infection and Lipid Metabolism in Hepatocytes: A Potential Target of Antiviral Therapy for Chronic Hepatitis B

**DOI:** 10.3389/fmicb.2021.636897

**Published:** 2021-03-11

**Authors:** Jiaxuan Zhang, Ning Ling, Yu Lei, Mingli Peng, Peng Hu, Min Chen

**Affiliations:** Key Laboratory of Molecular Biology for Infectious Diseases, Ministry of Education, Department of Infectious Diseases, Institute for Viral Hepatitis, The Second Affiliated Hospital of Chongqing Medical University, Chongqing, China

**Keywords:** hepatitis B virus, chronic hepatitis B, lipid metabolism, apolipoprotein, hepatic steatosis, metabolic signaling pathway, nuclear factors

## Abstract

Hepatitis B virus (HBV) is considered a “metabolic virus” and affects many hepatic metabolic pathways. However, how HBV affects lipid metabolism in hepatocytes remains uncertain yet. Accumulating clinical studies suggested that compared to non-HBV-infected controls, chronic HBV infection was associated with lower levels of serum total cholesterol and triglycerides and a lower prevalence of hepatic steatosis. In patients with chronic HBV infection, high ALT level, high body mass index, male gender, or old age was found to be positively correlated with hepatic steatosis. Furthermore, mechanisms of how HBV infection affected hepatic lipid metabolism had also been explored in a number of studies based on cell lines and mouse models. These results demonstrated that HBV replication or expression induced extensive and diverse changes in hepatic lipid metabolism, by not only activating expression of some critical lipogenesis and cholesterolgenesis-related proteins but also upregulating fatty acid oxidation and bile acid synthesis. Moreover, increasing studies found some potential targets to inhibit HBV replication or expression by decreasing or enhancing certain lipid metabolism-related proteins or metabolites. Therefore, in this article, we comprehensively reviewed these publications and revealed the connections between clinical observations and experimental findings to better understand the interaction between hepatic lipid metabolism and HBV infection. However, the available data are far from conclusive, and there is still a long way to go before clarifying the complex interaction between HBV infection and hepatic lipid metabolism.

## Introduction

Hepatitis B virus (HBV), a member of the *Hepadnaviridae* family, is one of the smallest enveloped animal DNA viruses and the pathogen to cause Hepatitis B. Although HBV vaccines and effective antiviral drugs have been available for more than 20 years, chronic HBV infection (CHB) remains a global public health problem, especially in Asia (Megahed et al., 2020). Increasing attention has been paid to the relationship between HBV infection and hepatic lipid metabolism recently. Several large-scale clinical studies have been conducted to explore the correlation of hepatic steatosis with CHB. Meanwhile, accumulated data have shown that HBV replication and expression could interact with some lipid metabolism-related transcription factors (TFs) and nuclear receptors (NRs) (Bar-Yishay et al., 2011; Wagner et al., 2011), including retinoid X receptor α (RXRα) (Reese et al., 2011), farnesoid X Receptor (FXR) (Mouzannar et al., 2019), estrogen-related receptors (ERs) (Wang et al., 2012), or peroxisome proliferator-activated receptors (PPARs) (Jiang et al., 2019). Simultaneously, cellular or animal studies have unveiled some mechanisms of how HBV affected the activity or expression of multiple factors involved in hepatic lipid metabolism, or vice versa (Lin et al., 2015; Wang C.C. et al., 2015; Shi et al., 2016; Suliman et al., 2019). Therefore, based on these recent clinical and basic studies, this article comprehensively reviewed the latest progress and opinions regarding HBV infection and hepatic lipid metabolism, and provided new insight into approaches for treating CHB.

## The Risk of Hepatic Steatosis in Patients With Chronic HBV Infection

Hepatic steatosis, also known as fatty liver disease, includes alcohol-related fatty liver disease (AFLD) and non-alcoholic fatty liver disease (NAFLD). So far, the prevalence of NAFLD in CHB patients remains unconfirmed. A meta-analysis conducted in Machado et al. (2011) showed that in CHB patients, the overall hepatic steatosis prevalence was 29.6%, which was lower than that in hepatitis C virus (HCV)-infected patients. Subsequently, one large-scale cross-sectional retrospective study involving 33,439 subjects in health checkup (Cheng et al., 2013) showed that the prevalence of fatty liver was lower in hepatitis B surface antigen positive (HBsAg^+^) populations (38.9%) than in the HBsAg^−^ group (44.5%), and HBsAg positivity was inversely correlated with fatty liver. These results were consistent with another large-sample prospective cohort study (Joo et al., 2017), which showed that HBsAg seropositivity was associated with a lower risk of developing NAFLD during an approximately 10-year follow-up of 83,339 non-NAFLD Korean adults with normal alanine aminotransferase (ALT) levels at baseline. Besides, a hospital-based case-control retrospective study showed that the negative correlation between HBV infection and fatty liver appeared only in patients with new-onset CHB (Zhong et al., 2018). In addition, these clinical studies had found that serum level of total cholesterol, triglycerides, HDL-C, or LDL-C in CHB patients was lower than that of non-HBV-infected controls (Wong et al., 2012; Cheng et al., 2013; Joo et al., 2017). A study involving only female subjects in health checkup reported that the prevalence of fatty liver exhibited no significant difference between HBV-infected patients and HBV-free subjects (Wang et al., 2019).

Apart from the comparison of the prevalence of hepatic steatosis between CHB patients and non-CHB controls, factors involved in NAFLD development among CHB populations were thoroughly investigated in some studies. Factors that had positive or negative influence on hepatic steatosis in CHB patients were shown in Supplementary Table 1. Among these factors, high BMI, male gender, old age, and high ALT levels were commonly reported to be positively associated with NAFLD in CHB patients (Peng et al., 2008; Shi et al., 2008; Zheng et al., 2010; Machado et al., 2011; Wong et al., 2012; Cheng et al., 2013; Wang et al., 2014, 2019; Enomoto et al., 2016; Charatcharoenwitthaya et al., 2017; Joo et al., 2017; Chen et al., 2018; Hui et al., 2018; Zhong et al., 2018; Zhu et al., 2019). However, there was no evident correlation between hepatic steatosis and HBeAg status or HBV DNA levels (Peng et al., 2008; Charatcharoenwitthaya et al., 2017).

Nevertheless, these clinical studies suggested that HBsAg positivity might be related to NAFLD’s low occurrence, while the status of liver inflammation and clinical or metabolic factors during disease progress would influence this relationship. Furthermore, differences in clinical study design, sample size, method for hepatic steatosis detection, or statistical method would deduce different conclusions. Clarifying the relationship between HBV infection and NAFLD development will require additional multi-center, large-sample, case-controlled clinical studies with hepatic steatosis assessment in liver biopsy samples.

## HBV-Induced Changes in Fatty Acid Metabolism

Fatty acid metabolism in the liver is controlled by a complex network of TFs and proteins (Alves-Bezerra and Cohen, 2017). In different cellular or mouse models, HBV replication or expression increased lipid biosynthesis-related factor sterol regulatory element-binding protein 1c (SREBP1c). SREBP1c activation upregulates the expression of lipogenic enzymes, such as fatty acid synthase (FAS), stearoyl-CoA desaturase (SCD), and acetyl-CoA carboxylase (ACC), and thus fatty acid synthesis (Wang Y. et al., 2015; Alves-Bezerra and Cohen, 2017). By the proteomic analysis of liver tissue from HBV transgenic mice, Yang et al. (2008) found that the expression of fatty acid-binding protein 5 (FABP5) and acetyl-CoA binding protein (ACBP), which were involved in fatty acid metabolism and synthesis, was significantly up-regulated compared to that in wild-type mice.

Levels of various types of lipids were also examined by mass spectrometry in different stages of chronic HBV infection. Arain et al. (2018) found that HBV patients showed significantly higher levels of serum saturated fatty acids (SFAs) or monounsaturated fatty acids (MUFAs) (myristic acid or palmitic acid), and lower levels of polyunsaturated fatty acids (PUFAs) (linoleic acid, eicosatrienoic acid, etc.). Moreover, a higher level of urine palmitic acid, stearic acid, oleic acid, or cholesterol was shown in CHB patients than in the control group (Dittharot et al., 2018), and a large difference between urine and serum metabolite profiles was found in the same CHB subject (Yang et al., 2016). In an AdHBV-infected primary rat hepatocyte model, metabolomic and transcriptomic datasets synergistically showed a noticeable increase in the long-chain FFA pool (Lamontagne et al., 2018). Furthermore, in a stable HBV-producing cell line HepG2.2.15, Li et al. (2015) found that the total fatty acid content was increased, but the essential fatty acids α-linolenic acid and linoleic acid were decreased.

Apart from reports that HBV increased lipogenesis, several other studies showed that HBV infection also increased fatty acid oxidation and reduced lipid droplets (LD) formation. Yasumoto et al. (2017) showed that the content of intracellular TGs and the average size of a single LD were significantly reduced in HBV-infected or transfected cells compared to control cells, and that was because of decreased levels of proteins involved in LD expansionary and lipid storage. Studies also showed that HBV replication increased adiponectin expression (a downstream gene of PPARγ) (Yoon et al., 2011). Adiponectin alleviated lipid accumulation by increasing carnitine palmitoyltransferase 1 (CPT1) activity, enhancing hepatic fatty acid oxidation, and reducing ACC and FAS activity (Wang M.D. et al., 2016; Fang and Judd, 2018).

Overall, these results from experimental studies indicated that HBV infection not only enhanced fatty acid synthesis and lipogenesis, but also increased fatty acid oxidation and lipolysis.

## Changes in Phospholipids Metabolism During HBV Infection

In addition, significant changes in phospholipids and sphingolipids were found in HBV expressed primary hepatocytes or cell lines and CHB patients. Li et al. (2015) found that HBV infection activated the expression of choline kinase alpha to upregulate phosphatidylcholine biosynthesis. Huang et al. (2019) found that serum phosphatidylcholine, phosphatidylethanolamine, and lysophosphatidic acid were increased in HBsAg^+^ patients compared to HBsAg^−^ individuals. This study also found that phosphatidylcholine synthesis-related enzymes, phosphoethanolamine transferase A and phospholipid phosphatase 1 (LPP1), were upregulated after HBV infection. Different pattern of phospholipid changes was displayed in different stages of chronic HBV infection. Schoeman et al. (2016) found that decreased phospholipids, lysophospholipids, and sphingomyelin in the immune tolerance phase of HBV infection compared to healthy controls. Also, Qu et al. (2014) found that serum sphingolipids in CHB patients were significantly higher than those in healthy controls, especially in patients with HBV-associated acute-on-chronic liver failure (HBV-ACLF), suggesting that serum sphingolipid levels might be associated with HBV infection and disease progression.

## HBV-Induced Changes in Cholesterol Metabolism

Changes in cholesterol metabolism were evidenced during HBV infection. Li et al. (2013) found in HepG2 cells that HBV replication increased the expression of LDLR and hydroxymethylglutaryl coenzyme A reductase (HMGCR), leading to an increase in cholesterol intake and synthesis. Wang et al. (2018) showed that in a mouse model with alcoholic fatty liver, HBV replication increased hepatic cholesterol deposition by enhancing the expression of the cholesterol synthesis-related genes SREBP2 and HMGCR. Rodgers et al. (2009) found a high level of 7-dehydrocholesterol (7-DHC) in hepatocytes with HBV replication. 7-DHC is the direct precursor of free cholesterol and the substrate of 7-dehydrocholesterol reductase (DHCR7). Xiao et al. (2020) found significantly increased DHCR7 expression in livers of hepatocellular carcinoma (HCC) patients with HBV infection. 7-DHC was also a biosynthesis precursor to vitamin D, thus increased DHCR7 promoted cholesterol synthesis while inhibited Vitamin D production (Prabhu et al., 2016). Therefore, vitamin D was frequently found to be insufficient in CHB patients, and effective antiviral therapy increased the vitamin D level (Hoan et al., 2018).

Bile acid synthesis from cholesterol in hepatocytes is essential for the digestion and absorption of lipids. Na^+^/taurocholate cotransporter (NTCP/SLC10A1), a sodium-dependent transporter responsible for the basolateral uptake of taurocholate, was found as an entry receptor for HBV (Yan et al., 2012). HBV entry would interfere with the normal function of NTCP for bile acid uptake from portal blood into hepatocytes (Eller et al., 2018). In HBV-infected human liver chimeric mice and liver biopsies of CHB patients, multiple changes in bile acid metabolism-related genes have been found, especially significantly upregulated expression of hCYP7A1 (Oehler et al., 2014).

## The Contribution of HBx Protein to Changes in Hepatic Lipid Metabolism

Among the seven HBV proteins, HBx is an essential viral regulatory protein and has been demonstrated to interact with various proteins located in the cytoplasm, nucleus, and mitochondria (Ma et al., 2011; Xie et al., 2014; Slagle and Bouchard, 2018). Therefore, the molecular mechanisms underlying lipid metabolism changes resulting from HBV infection have been mainly focused on the HBx protein. Several studies revealed that HBx protein overexpression upregulated gene expression and transcriptional activation of LXRα/β, SREBP1, C/EBPα, and PPARγ, which contributed to hepatic lipid synthesis (Na et al., 2009; Shieh et al., 2010; Yoon et al., 2011; You et al., 2013; Cui et al., 2014a,b; Wu et al., 2016; Xu et al., 2016; Bai et al., 2017; Wang et al., 2018). In addition to the direct regulation of metabolism-related proteins, HBx was reported to regulate the miRNAs expression, such as miR-384 or miR-205, which subsequently regulated downstream proteins and lipid metabolism (Cui et al., 2014a; Bai et al., 2017). Besides, Wu et al. (2016) showed that HBx also increased intracellular trafficking of fatty acid by inducing the expression of fatty acid-binding protein 1 (FABP1). Furthermore, some evidence suggested that HBx overexpression increased cholesterol level in hepatocytes (Cui et al., 2014b; Wang et al., 2018).

## The Effect of HBV on Apolipoprotein Metabolism in Hepatocytes

Lipids, including TG, PLs, and cholesterol, are combined with apolipoprotein for their transport and metabolism. Hepatocytes are the major sites of apolipoprotein synthesis and lipoprotein assembly, secretion, uptake, and catabolism. Some clinical studies in HBV-infected population have found significantly decreased levels of plasma ApoA1 (Jiang et al., 2014; Wang Y. et al., 2016; Cui et al., 2019), ApoA5 (Zhu et al., 2016; Cui et al., 2019), ApoB (Wang et al., 2011; Cui et al., 2019), and ApoC3 (Zhu et al., 2017; Cui et al., 2019), whereas dramatically increased level of serum ApoM or ApoE (Gu et al., 2011; Shen et al., 2016). These results from clinical studies were consistent with observations from several experimental studies. ApoA1 promoted cholesterol transfer from peripheral tissues to the liver and increased cholesterol metabolism (Chistiakov et al., 2016). In the HepG2.2.15 cell line, HBV upregulated DNA methyltransferase activity to hypermethylate the ApoA1 promoter, thereby inhibited ApoA1 mRNA and protein expression (Jiang et al., 2014; Wang Y. et al., 2016). Besides, the lipid-binding ability of ApoA1 was impaired by its interaction with HBx (Zhang et al., 2013a), and HBc protein inhibited the activity of the ApoA5 gene promoter and reduced its expression (Zhu et al., 2016). ApoB100 participated in the transfer of cholesterol to peripheral tissues, which was opposite to the function of ApoA1 (Morita, 2016). Kang et al. (2004) showed that HBx protein overexpression in hepatocytes decreased ApoB secretion, increased the intracellular levels of ApoB, TG, and cholesterol, and interfered with VLDL/LDL assembly or secretion. ApoC3 acted as an inhibitor of lipoprotein lipase (LPL), which was a crucial enzyme in TG lipoprotein catabolism (Larsson et al., 2017). A study (Zhu et al., 2017) showed that HBV inhibited the synthesis and secretion of ApoC3. Therefore, during chronic HBV infection, a decrease in ApoC3 expression would increase LPL activity, decrease VLDL synthesis and secretion, and increase TG decomposition.

ApoE acted as a ligand of LDLR or heparan sulfate proteoglycan (HSPG) and played a central role in the transport, metabolism, and homeostasis of cholesterol and other lipids (Getz and Reardon, 2018). HBV infection led to an increase in ApoE expression (Shen et al., 2015), and ApoE promoted HBV infection and production (Qiao and Luo, 2019). ApoM was expressed only in hepatocytes and renal tubular cells, and it promoted HDL (β-HDL) formation and increased cholesterol efflux from foam cells (Ren et al., 2015). Overexpression of ApoM showed to inhibit HBV replication in HepG2 cells (Gu et al., 2011). Also, the myristoylated pre-S1-domain of the HBV L-protein strongly interacted with apolipoprotein H, which was involved in activation of lipoprotein lipase, and inhibition of platelet prothrombinase activity (Stefas et al., 2001).

**FIGURE 1:**
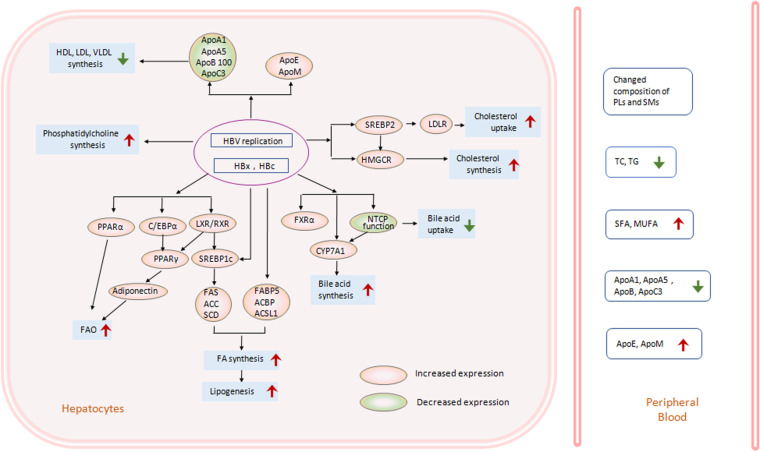
HBV-induced lipid metabolism changes in hepatocytes. HBV infection, replication, and expression in hepatocytes can change hepatic lipid metabolic pathways in many aspects. In HBV-transfected cell lines, HBV-transgenic mice or liver specimens from CHB patients, transcriptional factors (TF), and genes related to hepatic lipogenesis have changed much. Enhanced activation of LXR and SREBP1c increased downstream lipogenesis genes, including FAS, ACC, SCD, et al., and promoted free fatty acid (FFA) biosynthesis and lipogenesis (Kim et al., 2007; Yang et al., 2008; Zhang et al., 2013b; Wu et al., 2016; Xu et al., 2016; Bai et al., 2017; Wang et al., 2017). On the other hand, activation of LXR, C/EBP, PPARα and PPARγ can also increase FFA oxidation through adinonectin, or other ways (Yoon et al., 2011; Wang M.D. et al., 2016). Simultaneously, phosphatidylcholine synthesis was enhanced too (Huang et al., 2019). The key TF SREBP2 and enzyme HMGCR in the cholesterol synthesis pathway were stimulated by HBV replication or HBx protein, inducing increased cholesterol uptake and synthesis in hepatocytes (Li et al., 2013; Wang et al., 2018). The interaction between HBV and NTCP facilitated CYP7A1 expression and bile acid biosynthesis (Yan et al., 2012; Oehler et al., 2014; Eller et al., 2018). Moreover, HBV replication decreased the expression of several types of apolipoprotein (Apo), including ApoA1 (Zhang et al., 2013a; Jiang et al., 2014; Wang Y. et al., 2016), ApoA5 (Zhu et al., 2016), ApoB100 (Morita, 2016), or ApoC3 (Zhu et al., 2017), and repressed synthesis of HDL, LDL, VLDL in hepatocytes (Kang et al., 2004). While expression of ApoE (Shen et al., 2015) or ApoM (Gu et al., 2011) increased after HBV infection. Changes in hepatic lipid metabolism induced changed lipid levels in peripheral blood, including increased SFA and MUFA (Arain et al., 2018), decreased ApoA1, ApoA5, ApoB100, or ApoC3 (Wang et al., 2011; Jiang et al., 2014; Wang Y. et al., 2016; Zhu et al., 2016, 2017; Cui et al., 2019), or increased ApoE or ApoM (Gu et al., 2011; Shen et al., 2016). However, the level of TG and TC showed a decrease in CHB patients compared to normal controls (Wong et al., 2012; Cheng et al., 2013; Joo et al., 2017). The expression of genes in the red circle was increased, while that in the green circle was decreased. (ACBP, acetyl-CoA binding protein; ACC, acetyl-CoA carboxylase; ACSL, long-chain fatty acyl-CoA synthetase; Apo, apolipoprotein; C/EBPα, CCAAT/enhancer-binding protein; CYP7A1, cholesterol 7α-hydroxylase; FA, fatty acid; FABP, Fatty acid-binding protein; FAS, Fatty acid synthetase; HBV, hepatitis B virus; FAO, fatty acid oxidation; FXR, farnesoid X receptor; HBx, hepatitis B virus X protein; HBc, hepatitis B virus core protein; HDL, high density lipoprotein; HMGCR, hydroxymethylglutaryl coenzyme A reductase; LDL, low density lipoprotein; LDLR, low density lipoprotein receptor; LXR, liver X receptor; MUFA, monounsaturated fatty acid; NTCP, Na/taurocholate cotransporter; PL, phospholipid; PPAR, peroxisome proliferators-activated receptor; RXR, retinoid X receptor; SCD, stearoyl-CoA desaturase; SFA, saturated fatty acid; SM, Sphngomyelin; SREBP1c, sterol-regulatory element-binding protein1c; SREBP2, sterol regulatory element-binding protein 2; TC, total cholesterol; TG, triglyceride; VLDL, very low density lipoprotein).

These results indicated that HBV replication had complex effects on lipid metabolic pathways (Figure 1). However, most results were obtained from HBV-expressing mice or cell lines. Therefore, to reveal the exact mechanisms of effects of HBV on hepatic lipid metabolism, more data from liver samples of CHB patients are needed.

## Hepatic Lipid Metabolism Affecting the Life Cycle of HBV: Potential Targets for HBV Suppression

**FIGURE 2:**
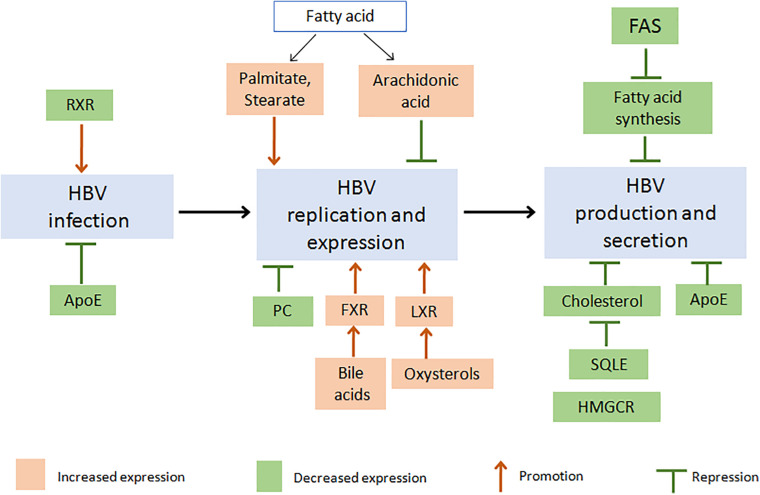
Changes in proteins or metabolites of lipid metabolism pathway affect the life cycle of HBV: potential targets for HBV suppression. Firstly, inhibition of FAS activity decreased fatty acid (FA) synthesis and then suppressed HBV production and secretion (Zhang H. et al., 2013; Cho et al., 2014; Okamura et al., 2016). An increasing level of different FA would induce a different effect on HBV, such as promotion of HBV replication and expression by palmitate or stearate (Cho et al., 2014), while suppression of HBV by arachidonic acid (Song et al., 2018). As cholesterol is the necessary component of HBV particle, inhibition of HMGCR or SQLE decreased cholesterol biosynthesis and suppressed HBV production (Lin et al., 2003; Bremer et al., 2009). Increased HBV replication could be induced through LXR activation by oxysterols or FXR activation by bile acid (Ramière et al., 2008; Kim et al., 2011; Zhao et al., 2018). And inhibition of ApoE decreased HBV infection, production, or secretion (Qiao and Luo, 2019). Besides, decreasing PC synthesis inhibited HBV replication (Tatematsu et al., 2011; Li et al., 2015), and inhibition of RXR increased HBV infection (Reese et al., 2011). Red or green boxes indicated increased or decreased levels of lipid and lipid metabolism-related proteins, respectively. (Apo, apolipoprotein; FA, fatty acid; FAS, Fatty acid synthetase; FXR, farnesoid X receptor; HBV, hepatitis B virus; HMGCR, hydroxymethylglutaryl coenzyme A reductase; LXR, liver X receptor; PC, phosphatidylcholine; RXR, retinoid X receptor; SQLE, Squalene monooxygenase).

As reviewed above, HBV infection induced a series of changes in the hepatic lipid metabolic pathways. On the other hand, changes in the levels of metabolic products or expression of metabolism-related proteins conversely affected the life cycle of HBV (Figure 2).

Zhang H. et al. (2013) showed that FAS, a key enzyme of fatty acid synthesis, was highly expressed in HBV transgenic mice, and silencing of FAS expression reduced HBV expression. Okamura et al. (2016) further found that FAS inhibitor decreased HBV particles’ production but not genomic replication. Cho et al. (2014) also uncovered that fatty acids (including palmitate, stearate, and oleate) increased HBx protein’s stability by preventing proteasome-dependent degradation. In addition to fatty acids, PC biosynthesis inhibition suppressed HBV replication and expression (Tatematsu et al., 2011; Li et al., 2015). Yoon et al. (2011) reported that adiponectin, although as a negative regulator of lipogenic genes (Combs and Marliss, 2014; Gamberi et al., 2018), acted directly on core particles to enhance HBV polymerase activity and HBV DNA replication. Moreover, Song et al. (2018) revealed that arachidonic acid (AA), a kind of PUFA, inhibited HBV infection. It might be because FFAs were potential ligands for toll-like receptor 4 (TLR4) and could activate the innate immune response (Mamedova et al., 2013; Han et al., 2018).

Cholesterol was necessary for the HBV envelope and played a critical role in HBV infectivity or viral particle secretion (Lin et al., 2003). Cholesterol depletion and cholesterol synthesis inhibitors, such as inhibitor of HMGCR or squalene cyclooxygenase, impaired secretion of either HBV viral or subviral particles in HBV-producing cell lines (Bremer et al., 2009). Moreover, a study found that oxygenated cholesterol derivatives (also named oxysterols, the endogenous agonists of LXR) increased the transcription and replication of HBV and reduced the anti-HBV effect of IFN (Kim et al., 2011). Interestingly, a recent study showed synthetic LXR agonists inhibited HBV replication and gene expression through CYP7A1 reduction in HBV-infected primary human hepatocytes, but this observation was not found in hepatocellular carcinoma cell line (Zeng et al., 2020). 25-hydroxycholesterol (25-HC) has been identified to block replication and entry of a broad range of viruses, such as VSV, HIV, EBOV, or ZIKA (Liu et al., 2013; Xiao et al., 2020; Zhao et al., 2020). However, there were few reports about the effect of 25-HC on HBV replication by now. Moreover, the combination use of VitD3 and IFNα enhanced the anti-HBV efficacy of IFNα (Farnik et al., 2013; He et al., 2016; Ko et al., 2020).

The effect of bile acid on HBV infection has received increasing attention. Studies showed that high expression of NTCP induced an increase in bile acid transport to hepatocytes, which promoted HCV and HBV infection by bile-acid-mediated repression of some interferon-stimulated genes (Verrier et al., 2016; Eller et al., 2018). Also, elevated intracellular bile acids activated FXRα, which activated HBV enhancer 2/core promoter and consequently enhanced HBV replication (Ramière et al., 2008; Zhao et al., 2018). In addition, FXRα silencing in HBV-infected HepaRG cells decreased the pool size and transcriptional activity of viral covalently closed circular (ccc)DNA (Mouzannar et al., 2019). Other TFs or NRs, such as activator protein-1 (AP-1), activating transcription factor 2 (ATF2), or cAMP-response element-binding protein (CREB), were also reported to be involved in the mechanisms underlying the upregulation of HBV replication by bile acids.

## Conclusion

Hepatitis B virus infection induces multiple changes in hepatic lipid metabolism, by increasing both lipid synthesis and lipolysis. These intracellular changes might be helpful to HBV replication through providing both material and energy. These observations also elucidate why HBV infection facilitates lipid synthesis while the risk of hepatic steatosis in CHB patients does not noticeably increase, and levels of serum TG and TC decrease. Moreover, targeting a certain lipid metabolism pathway would be a potential therapeutic way to chronic hepatitis B. Given that many studies are limited to cells and animal models and evidence is far from conclusive, more well-designed clinical studies are needed to elucidate the mechanism of this interaction and to discover metabolic targets that could suppress HBV replication or improve the anti-HBV effect of IFN or nucleot(s)ide analogs.

## Author Contributions

JZ, NL, and YL: conception and literature review. JZ and MC: writing of the original draft. MC and PH: review and editing of the manuscript. MC: funding acquisition. MP and PH: supervision of the manuscript. All authors have read and agreed to the published version of the manuscript.

## Conflict of Interest

The authors declare that the research was conducted in the absence of any commercial or financial relationships that could be construed as a potential conflict of interest.
