# Supplementary material for: Multifaceted Interaction Between Hepatitis B Virus Infection and Lipid Metabolism in Hepatocytes: A Potential Target of Antiviral Therapy for Chronic Hepatitis B
Source: Front Microbiol. 2021 Mar 11;12:636897. doi: 10.3389/fmicb.2021.636897 (PMC7991784; doi:10.3389/fmicb.2021.636897)
Supplement: Supplementary file 1 [file Table_1.DOCX]

**Supplementary Table 1. Representative clinical studies on risk of liver steatosis in patients with chronic HBV infection**

| **Study population Sample size (n)** | **diagnosis method of liver steatosis** | **Prevalence of  liver steatosis** | **Hazard ratio/OR of developing NAFLD  in patients with CHB (95% CI)** | **Factors positively associated  with liver steatosis** | **Factors negatively associated  with liver steatosis** | **Factors with no association  with liver steatosis** | **Main results** | **Reference** |
| --- | --- | --- | --- | --- | --- | --- | --- | --- |
| Meta-analysis CHB (n=4100) | liver biopsy | CHB:29.6% | 0.55 (0.45-0.67） (compares to CHC) | male gender,  BMI, obesity, DM, glycemia,  TG, TC, moderate alcohol consumption | HBV DNA load | aminotransferases, HBeAg, genotypes or hepatic histology,  necroinflammation or fibrosis | decreased risk of hepatic steatosis in HBV versus HCV patients | Machado et al., 2011 |
| Taiwan, China， health check-up,  CHB(HBsAg+) (n=3642) Control(HBsAg-) (n=29797) | ultrasonography | CHB:38.9% Overall:43.9% | NA | BMI, age,  WC, SBP,  FBG, TC, ALT, platelet counts | HBsAg | NA | CHB Patients were inversely associated with fatty liver disease than the general population, especially in older and obese patients | Cheng et al., 2013 |
| South Korea， Cohort study， health check-up， CHB(HBsAg+) (n=3926) Control(HBsAg-) (n=79413) | ultrasonography | CHB:40.6/1000 person-years Control:43.5/1000 person-years | 0.83 (0.73-0.94) | NA | NA | NA | HBsAg seropositivity  is associated with lower risk of developing NAFLD | Joo et al., 2017 |
| China, case-control study， CHB(HBsAg+) (n=3212) | liver biopsy | CHB:17.3% | NA | male gender, age | HBV DNA load,  intrahepatic HBsAg and HBcAg, HBeAg | NA | Hepatic steatosis in HBV  infected patients is negatively associated with intrahepatic expression of HBsAg | Wang et al., 2014 |
| Hong Kong, China， case-control study, CHB(HBsAg+) (n=1202) Steatosis(n=601)  No Steatosis(n=601) | controlled attenuation parameter (CAP) (measured by transient elastography) | CHB:56.6% (1548 CHB patients) | 0.859 (0.743-0.994) | central obesity,  MS, BMI,  platelet count | HBV DNA load | NA | Increasing hepatic steatosis  was independently associated with lower serum HBV DNA levels | Hui et al., 2017 |
| China, case-control study, CHB(HBsAg+,HBeAg-) (n=204) Steatosis(n=106)  No Steatosis(n=98) | liver biopsy | NA | NA | FI | NA | NA | HBeAg-negative chronic hepatitis B patients with hepatic steatosis had significantly lower HBV DNA load | Zheng et al., 2010 |
| China, case-control study, controls(n=2357) newly diagnosed NAFLD(n=631) | ultrasonography | NA | current HBV infection 0.64 (0.42-0.95) | NA | NA | age, sex,  BMI, current smoking, diabetes, ALT, FBG, TG | current but not past HBV infection is associated with a decreased risk of NAFLD in the Chinese population | Zhong et al., 2018 |
| China， case-control study, CHB(HBsAg+) (n=1915) | liver biopsy | CHB:14% | NA | BMI, TG, ApoB,  Uric acid, FBG | NA | HBV DNA load,  HBeAg | Hepatic steatosis  is associated with metabolic factors not viral ones | Shi et al., 2008 |
| China, Cohort study， health check-up, female, CHB(HBsAg+) (n=152) Control(HBsAg-) (n=1714) | ultrasonography | CHB:10.5％ Control:17％ | 0.656 (0.379-1.134) | NA | NA | NA | the incidence of fatty liver disease in HBV‑infected subjects was not significantly different from in non‑HBV‑infected subjects | Wang et al., 2019 |
| Thailand, case-control study, CHB(HBsAg+, HBV DNA level >2,000 IU/ml) (n=256) | liver biopsy | CHB:38% | NA | obese, BMI, hypertriglyceridaemia | HDL | HBeAg, genotype | Hepatic steatosis in HBV  infected patients is associated with metabolic syndrome but not viral factor | Charatcharoenwitthaya P et al., 2017 |
| China， Cohort study， CHB(HBsAg+) (n=2393) | liver biopsy | CHB:63.89/1000 person-years | NA | BMI, DM | NA | HBV DNA load,  HBeAg, age, gender | HBV carriers had an increased risk  of NAFLD for those overweight and obese, and with concurrent type 2 diabetes mellitus, and in the subgroup of participants with concurrent type 2 diabetes mellitus, HBV DNA levels were negatively associated with the development of NAFLD | Zhu et al., 2019 |
| Taiwan, China， health check-up,  CHB(HBsAg+) (n=50) Control(HBsAg-) (n=457) | ultrasonography | CHB:56% | NA | TG, BMI, HOMA-IR | NA | HBsAg, age, gender, ALT, TC, FBG | Chronic HBV infection  seems not to be associated with hepatic steatosis in HBV carriers | Wang et al., 2008 |
| Iran, case-control study, CHB(HBsAg+) (n=132) | liver biopsy | CHB:42.4% | NA | TG | NA | age, sex,  HBeAg,  HBV DNA load,  TC, AST, ALT | metabolic host factors rather than viral factors responsible for the presence of hepatic steatosis in HBV infected patients | Minakari M et al., 2009 |
| India, case-control study, CHB(HBsAg+)(n=350) | liver biopsy | CHB:33.7% | NA | age, gender, BMI,  TG, TC, FI | HBV DNA load | NA | Hepatic steatosis is associated with host metabolic factors, especially serum triglyceride levels, whereas HBV DNA level negatively correlated with hepatic steatosis | Rastogi A et al., 2011 |
| China, Cohort study, children(0-18 years) CHB(n=560) CHB+NAFLD(n=62) NAFLD(n=143) | liver biopsy | NA | NA | BMI, Globulin,  Prealbumin | Creatinine,  HBV DNA load | NA | an inverse association between  CHB and NAFLD reciprocally existed in pediatric population | Wang L et al., 2019 |
